# Early de-escalation of empiric antibiotic therapy in neutropenic fever: a single-center, retrospective study

**DOI:** 10.1017/ash.2025.10208

**Published:** 2025-10-30

**Authors:** Zachary Mostel, Michelle Evans, Douglas Tremblay, Meenakshi Rana, Samantha E. Jacobs, John Mascarenhas, Alla Keyzner, Daniel Park, Risa Fuller

**Affiliations:** 1 Division of Infectious Disease, Department of Medicine, Icahn School of Medicine at Mount Sinai, New York, USA; 2 Tisch Cancer Institute, Icahn School of Medicine at Mount Sinai, New York, USA; 3 Department of Pharmacy, Icahn School of Medicine at Mount Sinai, New York, USA

## Abstract

We implemented a policy to discontinue empiric antibiotics after 48 hours of defervescence in neutropenic fever with no identified clinical/microbiologic infection. Among patients with acute myeloid leukemia or hematopoietic stem cell transplant, early de-escalation was not associated with increased subsequent infection, decompensation, or mortality, supporting its safety and feasibility.

## Introduction

**Figure 1.**
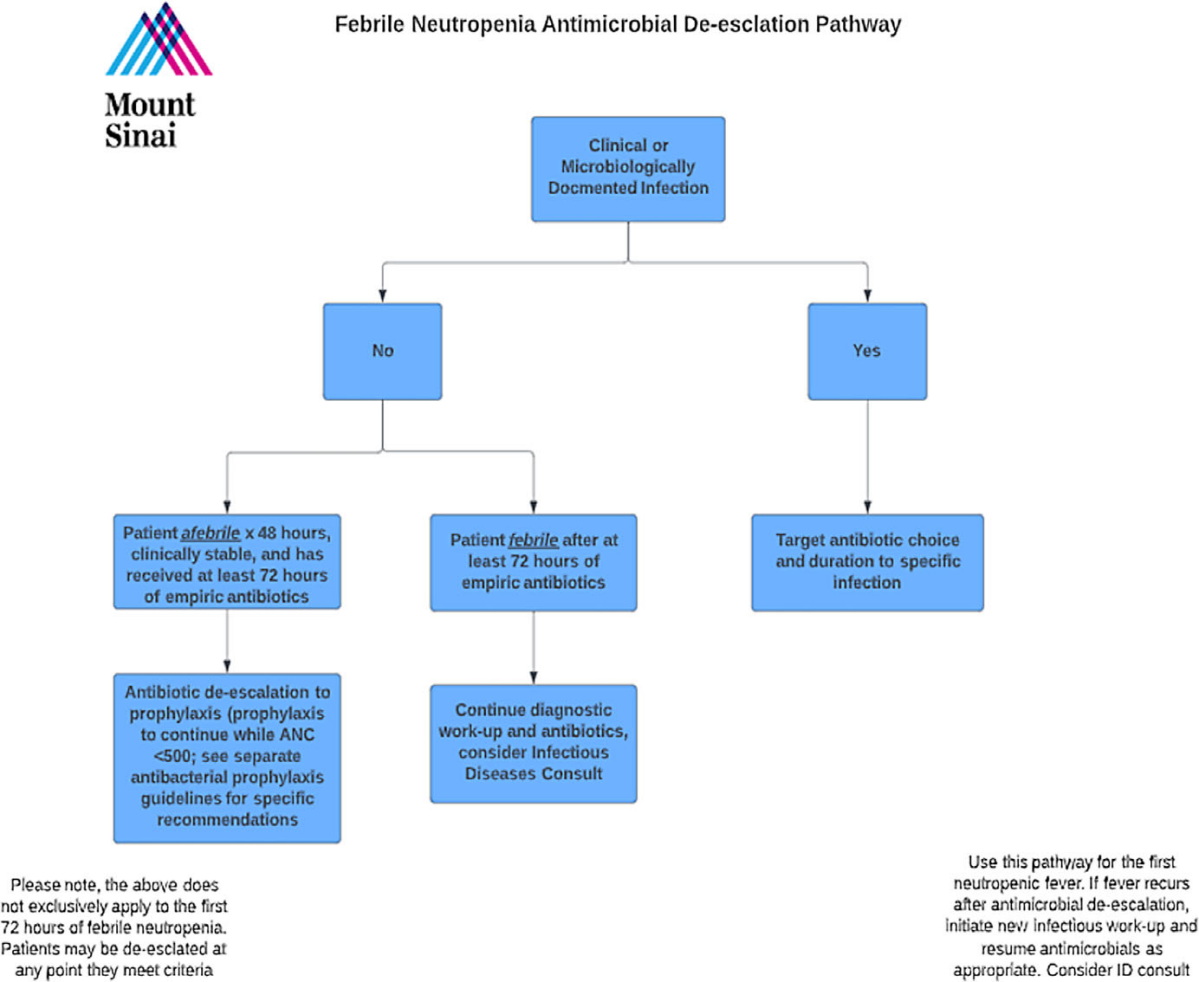
Febrile neutropenia antimicrobial de-escalation pathway. Abbreviations: ANC, absolute neutrophil count, ID, infectious diseases.

The duration of empiric antibiotic therapy for neutropenic fever (NF) without an infectious source remains controversial, with variable recommendations across clinical guidelines.^1,2^ The Fourth European Conference on Infections in Leukemia (ECIL-4) recommended discontinuing empiric antibiotics at 72 hours in clinically stable, afebrile patients.^3^ A recent review found seven studies that have evaluated early antibiotic de-escalation (EAD) using this approach and have found no increased rate of subsequent infection or clinical decompensation; these studies included a variety of empiric antipseudomonal agents.^4–11^ In March 2023, we implemented a new policy recommending cessation of empiric antibiotics or de-escalation to prophylaxis in patients with acute myeloid leukemia (AML) or recipients of a hematopoietic stem cell transplant (HSCT) who had NF without an identifiable infectious source, had been afebrile for at least 48 hours, and had received a minimum of 72 hours of empiric therapy, regardless of absolute neutrophil count (ANC) (Figure 1). This policy change followed an internal review of EAD in patients with AML, which found no increase in adverse events, clinical decompensation, or mortality.^12^ The purpose of this study was to evaluate the impact of a revised institutional policy for EAD on the duration of empiric antibiotic therapy during NF in this patient population. Additionally, we compared clinical outcomes before and after its implementation.

## Methods

We conducted a retrospective, quasi-experimental, single-center observational study of adults (≥18 yr) admitted to an oncology unit from March 2021 to August 2022 (preintervention group) and March 2023 to August 2024 (EAD/postintervention group). Eligible patients had NF, defined as a single temperature ≥38.3°C or ≥38.0°C sustained for ≥1 hour with an ANC ≤0.5 x 10^9^/L, in the setting of recent chemotherapy for AML or conditioning for HSCT, and received cefepime as empiric therapy for NF. Exclusion criteria included a documented clinical (physical examination or radiographic) or microbiologic infection or failure to de-escalate per protocol in the EAD group, despite being eligible per clinical criteria. Subgroup analyses were performed based on duration of neutropenia: short (≤7 d) and prolonged (≥8 d). Universal levofloxacin prophylaxis was routinely administered to all HSCT recipients while neutropenic and was newly implemented for patients with AML and neutropenia during the intervention period; there were no other changes in supportive care practices during either study period.

The primary outcome was duration of empiric antibiotic therapy for NF. Secondary outcomes included total duration of broad-spectrum antibiotic use during neutropenia, duration of antibiotic therapy after defervescence, subsequent infections following antibiotic de-escalation, clinical decompensation (ICU admission or vasopressor use), in-hospital mortality, and 30-day re-admission. Broad-spectrum antibiotics included any parenteral antibiotic used for hospital-onset infections and those with coverage of resistant gram-positive infections in accordance with definitions from National Healthcare Safety Network (NHSN).^13^ Descriptive statistics were used for data analysis. Comparisons between groups were performed using the Wilcoxon rank-sum test for continuous variables, and Fisher’s exact or Pearson’s χ2 test for categorical variables. Continuous variables were reported as median (interquartile range) unless otherwise specified. Statistical analysis was conducted using RStudio (version 2024.12.1 + 563). The institutional review board of the Icahn School of Medicine at Mount Sinai approved this study.

## Results

A total of 529 episodes of NF were identified. There were 149 episodes of NF in the preintervention cohort and 123 episodes in the EAD cohort among 252 total patients after excluding those with documented infection (*n* = 240) and non-adherence to the de-escalation protocol (*n* = 17). We excluded eligible patients who were not de-escalated, as the goal was to assess the impact of antimicrobial de-escalation. Baseline characteristics—including age, sex, race, clinical characteristics, subgroup distribution, and duration of neutropenia—were similar between cohorts (supplementary tables 1, 2, 3, 4).

The duration of empiric antibiotic therapy for NF decreased significantly from 6 (4–8) days in the preintervention group to 4 (3–5) days in the EAD group (*P* < .001). This reduction was observed in both the short (4 [3.5–6] vs 3 [3–4] days, *P* < .001) and prolonged neutropenia subgroups (7 [5–11] vs 4 [4–6] days, *P* < .001). The duration of broad-spectrum antibiotic use during neutropenia decreased significantly among patients with prolonged neutropenia (10 [6–16] vs 8 [5–12] days, *P* = .026); no statistically significant difference was observed in the overall cohort (5 [3–10] vs 4 [3–8] days, *p* = .12) and there was no difference among those with short-duration neutropenia (3 [2–5] vs 3 [3–4], *p* = 0.40). The duration of antibiotic therapy following defervescence decreased from 3 (2–5) to 2 (1–2) days (*P* < .001).

There were no significant differences between preintervention and EAD groups in rates of subsequent infection (29 [19%] vs 26 [21%], *P* = .70), clinical decompensation (5 [3.4%] vs 10 [8.1%], *P* = .086), decompensation attributed to infection (2 [1.3%] vs 4 [3.2%], *P* > .90), or in-hospital mortality (2 [1.3%] vs 3 [2.4%], *P* = .70). Among isolated organisms from subsequent bloodstream infections, 8 of 13 (62%) in the preintervention group and 14 of 18 (78%) in the EAD group were not susceptible to fluoroquinolones. Clinical decompensation predominantly occurred in patients with prolonged neutropenia (mean duration, 23.6 d; range, 5–36), who also received extended courses of empiric antibiotic therapy (mean, 8.9 d; range, 3–28) and broad-spectrum antibiotics during neutropenia (mean, 15.4 d; range, 4–44).

One patient in the EAD group died from clinically diagnosed pneumonia. In addition, there were no differences in mean hospital length of stay (21 [17–33] vs 23 [16–37] days, *P* = .50), rates of recurrent NF after cessation of empiric antibiotic therapy (47 [32%] vs 35 [28%], *P* = .60), or rehospitalization at 30 days (32 [21%] vs 33 [27%], *P* = .30) between the preintervention and EAD groups.

## Discussion

This study demonstrates a significant reduction in the duration of empiric antibiotic therapy for NF, with particular benefit for patients with prolonged neutropenia who historically receive extended courses of broad-spectrum antibiotics. Importantly, this reduction was not associated with increased rates of subsequent infection, clinical decompensation, or in-hospital mortality. Clinical decompensation was largely due to non-infectious reasons, and these patients typically received longer courses of broad-spectrum antibiotics regardless of NF or EAD.

Strengths of the study include the sole examination of a population at higher risk for decompensation due to infection during NF as well as its real-world applicability for both physicians and patients. The exclusive use of cefepime as empiric therapy allows for a more controlled assessment of EAD without the confounding influence of other antipseudomonal agents. The inclusion of agents such as piperacillin-tazobactam, carbapenems, aminoglycosides, or aztreonam—often selected based on provider preference or specific clinical scenarios (eg, suspected intra-abdominal source, colonization with multidrug-resistant organisms, or penicillin allergy)—can introduce provider bias. While prior studies have examined antimicrobial de-escalation in NF broadly, we demonstrated a significant benefit among patients with prolonged neutropenia, representing an important contribution to the literature.

However, the study has several limitations. The sample size and effect size were small, limiting statistical power; a larger cohort is needed to confirm these findings. Its retrospective, single-center, open-label, non-randomized design introduces potential for selection bias. Our analysis did not account for non–malignancy-related comorbidities, which have been included in other studies; this difference may limit the comparability of our findings to previously published cohorts. The generalizability of our findings may be limited in centers where local epidemiology precludes the use of cefepime therapy or fluoroquinolone prophylaxis; however, even in our large tertiary center with considerable resistance, these agents remain in use as empiric therapy in the context of stewardship. The implementation of universal levofloxacin prophylaxis during the intervention period for patients with AML and neutropenia may have influenced clinical outcomes and providers’ willingness to de-escalate empiric antibiotics. In addition, we excluded eligible patients who were not de-escalated, as our objective was to assess the impact of antimicrobial de-escalation. Retrospective review did not identify reasons for non–de-escalation, highlighting a further opportunity for antimicrobial stewardship. Lastly, clinical care pathways remained unchanged between the two study periods, which were temporally close, though supportive care for febrile neutropenia may have improved over time. Despite these limitations, the findings support the ECIL-4 recommendations and contribute to the growing body of literature suggesting that EAD in NF without a documented source is both safe and effective at reducing the duration of empiric therapy.

In a population highly vulnerable to infectious complications and antimicrobial resistance, antimicrobial stewardship is critical. Collaboration among infectious diseases, oncology, and pharmacy services is essential to the successful implementation and sustained adherence to changes in practice. These results add to the evidence that shorter courses of empiric antibiotics can be safely utilized in select patients with NF, without compromising clinical outcomes.

## Supporting information

10.1017/ash.2025.10208.sm001 Mostel et al. supplementary material Mostel et al. supplementary material

## Supplementary material

The supplementary material for this article can be found at https://doi.org/10.1017/ash.2025.10208.

## References

[1] Freifeld AG , Bow EJ , Sepkowitz KA , et al. Clinical practice guideline for the use of antimicrobial agents in neutropenic patients with cancer: 2010 update by the Infectious Diseases Society of America. Clin Infect Dis 2011;52:e56– e93. 21258094 10.1093/cid/cir073

[2] Klastersky J. Management of fever in neutropenic patients with different risks of complications. Clin Infect Dis 2004;39:S32– S37. 15250018 10.1086/383050

[3] Averbuch D , Orasch C , Cordonnier C , et al. European guidelines for empirical antibacterial therapy for febrile neutropenic patients in the era of growing resistance: summary of the 2011 4th European Conference on Infections in Leukemia. Haematologica 2013;98:1826– 1835. 24323983 10.3324/haematol.2013.091025 PMC3856957

[4] Stohs EJ , Abbas A , Freifeld A. Approach to febrile neutropenia in patients undergoing treatments for hematologic malignancies. Transpl Infect Dis 2024;26:e14236. 38349035 10.1111/tid.14236

[5] Aguilar-Guisado M , Espigado I , Martín-Peña A , et al. Optimisation of empirical antimicrobial therapy in patients with haematological malignancies and febrile neutropenia (How long study): an open-label, randomised, controlled phase 4 trial. Lancet Haematol 2017;4:e573– e583. 29153975 10.1016/S2352-3026(17)30211-9

[6] Le Clech L , Talarmin JP , Couturier MA , et al. Early discontinuation of empirical antibacterial therapy in febrile neutropenia: the ANTIBIOSTOP study. Infect Dis (Lond) 2018;50:539– 549. 29451055 10.1080/23744235.2018.1438649

[7] Verlinden A , Jansens H , Goossens H , et al. Safety and efficacy of antibiotic de-escalation and discontinuation in high-risk hematological patients with febrile neutropenia: a single-center experience. Open Forum Infect Dis 2021;9:ofab624. 35146042 10.1093/ofid/ofab624 PMC8826378

[8] Paret R , Le Bourgeois A , Guillerm G , et al. Safety and risk of febrile recurrence after early antibiotic discontinuation in high-risk neutropenic patients with haematological malignancies: a multicentre observational study. J Antimicrob Chemother 2022;77:2546– 2556. 35748614 10.1093/jac/dkac190

[9] Rearigh L , Stohs E , Freifeld A , Zimmer A. De-escalation of empiric broad spectrum antibiotics in hematopoietic stem cell transplant recipients with febrile neutropenia. Ann Hematol 2020;99:1917– 1924. 32556455 10.1007/s00277-020-04132-0 PMC7340662

[10] Contejean A , Abbara S , Chentouh R , et al. Antimicrobial stewardship in high-risk febrile neutropenia patients. Antimicrob Resist Infect Control 2022;11:52. 35346373 10.1186/s13756-022-01084-0 PMC8961889

[11] la Martire G , Robin C , Oubaya N , et al. De-escalation and discontinuation strategies in high-risk neutropenic patients: an interrupted time series analyses of antimicrobial consumption and impact on outcome. Eur J Clin Microbiol Infect Dis 2018;37:1931– 1940. 30051357 10.1007/s10096-018-3328-1

[12] Fuller R , Moshier E , Jacobs SE , et al. Practicing antimicrobial stewardship: de-escalating antibiotics in patients with acute myeloid Leukemia and neutropenic fever. Open Forum Infect Dis 2020;7:ofaa138. 32420406 10.1093/ofid/ofaa138 PMC7216765

[13] Centers for Disease Control and Prevention. National Healthcare Safety Network (NHSN) Patient Safety Component Manual. Atlanta, GA: Centers for Disease Control and Prevention. https://www.cdc.gov/nhsn/pdfs/pscmanual/pcsmanual-current.pdf. Accessed September 21, 2025.

