# Supplementary material for: Early de-escalation of empiric antibiotic therapy in neutropenic fever: a single-center, retrospective study
Source: Antimicrob Steward Healthc Epidemiol. 2025 Oct 30;5(1):e292. doi: 10.1017/ash.2025.10208 (PMC12616562; doi:10.1017/ash.2025.10208)
Supplement: Mostel et al. supplementary material [file S2732494X25102088sup001.docx]

Supplementary Table 1. Population Characteristics of Study Cohort

Abbreviations: EAD = Early Antibiotic De-escalation

Supplementary Table 2. Underlying Diagnoses of Study Cohort

Abbreviations: ALL = Acute Lymphoblastic Leukemia, AML = Acute Myeloid Leukemia, CLL = Chronic Lymphocytic Leukemia, CML = Chronic Myeloid Leukemia, EAD = Early Antibiotic De-escalation, MDS = Myelodysplastic Syndrome

Supplementary Table 3. Clinical Characteristics of Study Cohort

Abbreviations: AML = Acute Myeloid Leukemia, EAD = Early Antibiotic De-escalation, HSCT = Hematopoietic Stem Cell Transplant

Supplementary Table 4. Duration of Neutropenia of Study Cohort

Abbreviations: EAD = Early Antibiotic De-escalation, IQR = Interquartile Range
